# Perioperative Elevation in Cell-Free DNA Levels in Patients Undergoing Cardiac Surgery: Possible Contribution of Neutrophil Extracellular Traps to Perioperative Renal Dysfunction

**DOI:** 10.1155/2016/2794364

**Published:** 2016-11-02

**Authors:** Yu Qi, Tokujiro Uchida, Mamoru Yamamoto, Yudai Yamamoto, Koji Kido, Hiroyuki Ito, Nagara Ohno, Miho Asahara, Yoshitsugu Yamada, Osamu Yamaguchi, Chieko Mitaka, Makoto Tomita, Koshi Makita

**Affiliations:** ^1^Department of Anesthesiology, Tokyo Medical and Dental University, Graduate School of Medical and Dental Sciences, 1-5-45 Yushima, Bunkyo-ku, Tokyo 113-8519, Japan; ^2^Department of Anesthesiology, The University of Tokyo, Graduate School of Medicine, Tokyo, Japan; ^3^Department of Critical Care Medicine, Yokohama City University Medical Center, Kanagawa, Japan; ^4^Clinical Research Center, Tokyo Medical and Dental University Hospital of Medicine, Tokyo, Japan

## Abstract

*Background*. This study aimed to determine the perioperative change in serum double-strand DNA (dsDNA) as a marker potentially reflecting neutrophil extracellular trap concentration in samples from patients undergoing cardiac surgery and to analyze a relationship between serum dsDNA concentrations and perioperative renal dysfunction.* Methods*. Serum dsDNA concentrations in samples that were collected during a previously conducted, prospective, multicenter, observational study were measured. Eighty patients undergoing elective cardiac surgery were studied. Serum samples were collected at baseline, immediately after surgery, and the day after surgery (POD-1).* Results*. Serum dsDNA concentration was significantly increased from baseline (median, 398 ng/mL [interquartile range, 372–475 ng/mL]) to immediately after surgery (median, 540 ng/mL [437–682 ng/mL], *p* < 0.001), and they were reduced by POD-1 (median, 323 ng/mL [256–436 ng/mL]). The difference in serum creatinine concentration between baseline and POD-1 was correlated with dsDNA concentration on POD-1 (*r*_*s*_ = 0.61, *p* < 0.001).* Conclusions*. In patients undergoing cardiac surgery, serum dsDNA concentration is elevated postoperatively. Prolonged elevation in dsDNA concentration is correlated with perioperative renal dysfunction. Further large-scale studies are needed to determine the relationship between serum concentration of circulating dsDNA and perioperative renal dysfunction.

## 1. Introduction

Cell-free double-strand DNA (dsDNA) is thought to be one of danger associated molecular patterns, and its plasma concentration can be elevated in patients with sepsis [1, 2] and multiple trauma [3]. Among multiple sources of cell-free dsDNA including necrotic cells and apoptotic cells, neutrophil might be an important source, because activated neutrophils release dsDNA-based structures, called neutrophil extracellular traps (NETs), which forms fibrous structures to trap circulating microorganisms and kills them by antimicrobial proteins that are derived from neutrophil granules and the cytoplasm [4–6]. However, excessive release of NETs into plasma results in endothelial injury, which may result in tissue injury in major organs [6, 7]. Previous studies have shown that circulating cell-free dsDNA concentration reflects the amount of NET formation in the blood [1, 3, 8], and in patients with multiple trauma, circulating cell-free dsDNA concentration reflects the severity of injury [3]. Major surgery has some common characteristics with trauma, including tissue damage, inflammation, and activation of the endothelium and immune cells. However, the perioperative change in dsDNA concentration and its contribution to perioperative organ dysfunction have not been determined yet.

The objective of this study was to analyze perioperative changes in serum dsDNA concentration, as a marker reflecting NET formation, in patients undergoing cardiac surgery. To understand the kinetics of dsDNA, activity and antigen concentration of deoxyribonuclease-1 (DNase-I) were also studied. To determine the contribution of neutrophil activation to increased serum dsDNA concentration, we measured the concentration of S100A12 as a marker of neutrophil activation [9] and examined the correlation between S100A12 and dsDNA concentration. Finally, we investigated whether renal dysfunction is related to an elevation in dsDNA concentration. For these purposes, we studied clinical data and serum samples from a previously conducted multicenter, observational study for biomarkers of respiratory failure after cardiovascular surgery [10, 11].

## 2. Methods

### 2.1. Study Design

We studied clinical data and biological samples which were collected in a previously conducted study for biomarkers for respiratory failure after cardiac surgery [10, 11]. The original study is registered in the University Hospital Medical Information Network (UMIN) Clinical Trials Registry (ID: UMIN000010674; http://www.umin.ac.jp/ctr/index/htm/), and the details and results have been previously published in full [10, 11]. Briefly, a total of 87 patients undergoing cardiac surgery were enrolled in the original study. Serum concentrations of sRAGE and angiopoietin-2 were found to be elevated postoperatively, and the range in elevation was associated with respiratory failure morbidity after cardiac surgery. Three university hospitals participated in this study, and approval for the protocol was obtained from each institutional review board for clinical studies (Institutional Review Board, Clinical Research Center, Tokyo Medical and Dental University Hospital; Graduate School of Medicine and Faculty of Medicine, The University of Tokyo Research Ethics Committee; General Administration Division General Affairs Section, Yokohama City University Medical Center). Preoperative exclusion criteria were as follows: (i) age < 20 years; (ii) history of myocardial infarction within 30 days before the day of surgery; (iii) preoperative diagnosis of pneumothorax; (iv) diagnosis of chronic obstructive pulmonary disease; (v) neuromuscular disease; (vi) morbid obesity (body mass index > 40 kg/m^2^); and (vii) systemic connective tissue disease. Postoperative exclusion criteria were as follows: (i) severe neurological complications requiring mechanical ventilation; (ii) the need for an extracorporeal circuit because of cardiac failure; and (iii) patients whose pulmonary artery occlusion pressure was >18 mmHg in the first postoperative week. As an additional exclusion criterion, patients who were treated with hemodialysis before surgery were excluded to study the relationship between serum dsDNA concentration and perioperative renal dysfunction. Each patient provided written informed consent. Details of clinical management (anesthesia, steroid use, and postoperative mechanical ventilation) are described in the report of the original study [10, 11]. Criteria for diagnosis of acute kidney injury (AKI) were based on the Acute Kidney Injury Network classification system [12].

### 2.2. Measurements of Serum dsDNA Concentration

Three patients who were treated with hemodialysis and four patients with limited availability of serum were excluded. Therefore, 80 patients were included in the current study. Serum samples were collected (i) immediately after induction of general anesthesia (baseline), (ii) immediately after surgery (PostOp), and (iii) the day after surgery (POD-1) [10, 11]. Serum dsDNA concentration was quantified by the method used in previous studies [3, 13], using Quant-iT PicoGreen dsDNA Reagent (#P7581, Molecular Probes, Eugene, OR, USA). Fluorescence was measured by the GloMax Fluorometer (Promega Japan) with filter setting of 485 nm (excitation) and 538 nm (emission).

### 2.3. Enzyme-Linked Immunosorbent Assay (ELISA) for Biomarkers and DNase-I Antigen

Commercially available ELISA kits were used to measure serum concentrations of S100A12 (S100A12/EN-RAGE ELISA Kit; Circulex, Nagano, Japan), serum neutrophil gelatinase-associated lipocalin (NGAL) concentration (DLCN20, R&D Systems, Minneapolis, MN, USA), the antigen of DNase-I (Human Deoxyribonuclease I ELISA Kit; Cusabio, Hubei, China), anti-nucleosome antibody concentration (Anti-Nucleosome, ELISA Kit; Orgentec Diagnostika, Mainz, Germany), and perinuclear anti-neutrophil cytoplasmic antibody (pANCA) concentration (ANCA-P ELISA Kit; Abnova, Taipei City, Taiwan). Data for PostOp serum interleukin- (IL-) 8, N-terminal pro-B-type natriuretic peptide (NT-proBNP), and troponin-T concentrations were obtained from the same samples of 80 patients from the results of the original study [10].

### 2.4. DNase Activity Assay

We used the ORG590 DNase Activity ELISA (Orgentec Diagnostika) with some modifications for the measurement of DNase activity. Serum samples (100 *µ*L) without dilution were used for measurements. The results were calculated as % activity reduction/mL (%AR) from the calibration curve and this was plotted with the results of six calibrators of defined concentration. Finally, DNase activity in mKuU/mL was calculated from %AR values using the regression formula based on the data that were shown in the product insert. We could not measure one sample from a patient undergoing valve surgery because of a shortage of sample. Therefore, we measured samples from 79 patients.

### 2.5. Statistical Analyses

All statistical analyses were performed using STATA/IC software (version 14, StataCorp, College Station, TX, USA). We used the Mann–Whitney* U* test for two-group comparisons. For intergroup comparisons, we used the chi-square test for categorical data and the Kruskal–Wallis test for numerical data which are not following a normal distribution. In the latter case, post hoc multiple comparisons were analyzed with Steel–Dwass's test. We used Spearman's test for correlation analyses and the correlation was graded depending on the *r*_*s*_ value as follows: *r*_*s*_ ≥ 0.4 and <0.7 as a moderate correlation and *r*_*s*_ ≥ 0.2 and <0.4 as a weak correlation. We analyzed contributing factors to the morbidity of AKI by multiple logistic regression analysis, using variables selected by univariate analyses as possible confounding factors. These factors consisted of baseline creatinine concentration, type of surgery (on-pump or off-pump), dose of red cell transfusion, PostOp IL-8, troponin-T, NT-proBNP concentrations, and duration of surgery. In these multivariable tests, backward elimination was performed at *p* > 0.15. To assess discrimination ability of logistic regression models, area under the curve of receiver operating characteristic curve (AUROC) was calculated for each model. Statistical significance was defined as *p* < 0.05.

## 3. Results

Baseline demographic data and clinical variables are shown in Table 1. Fifty-one patients underwent on-pump surgery (19 patients underwent aortic surgery; 32 patients underwent valve surgery) and 29 patients underwent off-pump surgery (29 patients underwent coronary artery bypass grafting). The duration of surgery was significantly longer in the on-pump surgery group compared with the off-pump group (*p* < 0.001). Significantly more transfusions were required in the on-pump surgery group compared with the off-pump surgery group (*p* = 0.001 for fresh frozen plasma; and *p* = 0.001 for platelet transfusion, Table 1). One patient died on POD-24 and another patient died on POD-57.

### 3.1. Perioperative Changes in dsDNA Concentration

**Figure 1:**
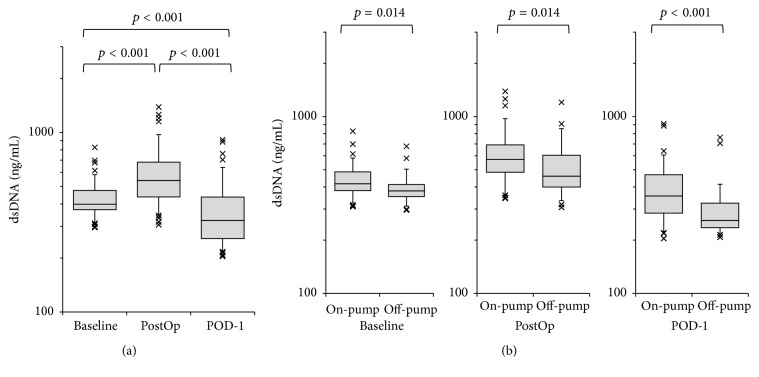
Perioperative changes in serum circulating double-strand DNA (dsDNA) concentration. (a) Perioperative changes in dsDNA concentration in the total cohort (*n* = 80). Measurements were performed at baseline, immediately after surgery (PostOp), and the day after surgery (POD-1). (b) Comparison of serum dsDNA concentration between on-pump (*n* = 51) and off-pump surgery (*n* = 29).

The perioperative changes in serum dsDNA concentration in patients who underwent cardiac surgery are shown in Figure 1(a). In the total cohort, PostOp serum dsDNA concentration was significantly higher than that at baseline (baseline median 398 ng/mL, interquartile range [IQR] 372–475 ng/mL versus PostOp median 540 ng/mL, IQR 437–682 ng/mL, *p* < 0.001) and had fallen significantly by POD-1 (median 323 ng/mL, IQR 256–436 ng/mL, Figure 1(a)). The off-pump surgery group had significantly lower serum dsDNA concentration than the on-pump surgery group at all three time points (*p* = 0.014 at baseline, *p* = 0.014 immediately after surgery, and *p* < 0.001 on POD-1, Figure 1(b)). There were eight patients mechanically ventilated more than 3 days, and these patients showed significantly higher concentration of serum dsDNA on POD-1 (median 477 ng/mL, IQR 367–608 ng/mL) than those extubated by POD-3 (median 315 ng/mL, IQR 215–419 ng/mL, *p* = 0.017).

**Figure 2:**
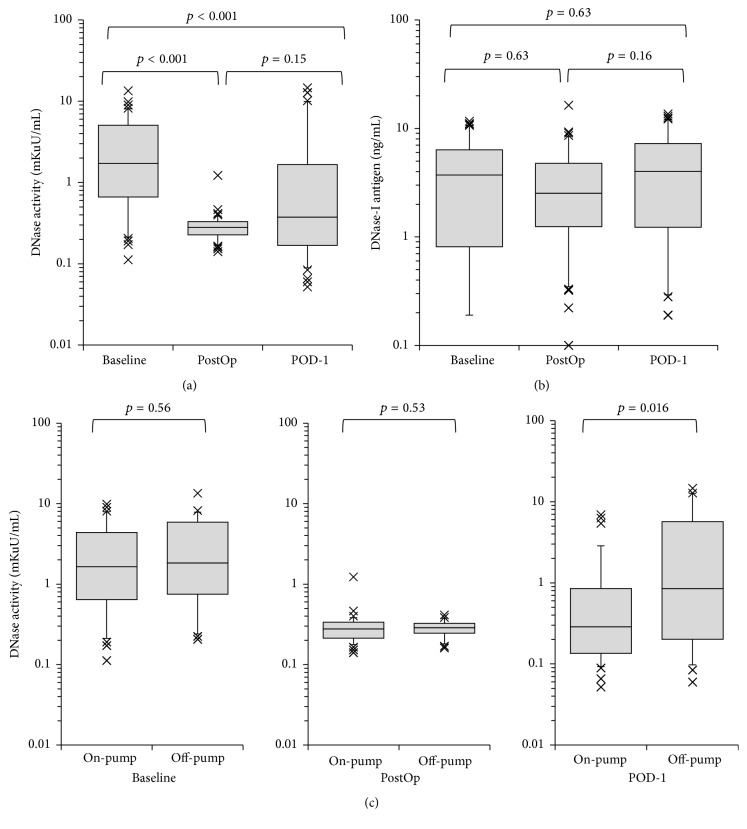
Perioperative changes in serum deoxyribonuclease (DNase) concentration. (a) Perioperative change in the activity of DNase in the total cohort. (b) Perioperative change in antigen concentration of DNase-I in the total cohort (*n* = 80). (c) Comparison of the activity of DNase between on-pump (*n* = 51) and off-pump surgery (*n* = 29).

**Figure 3:**
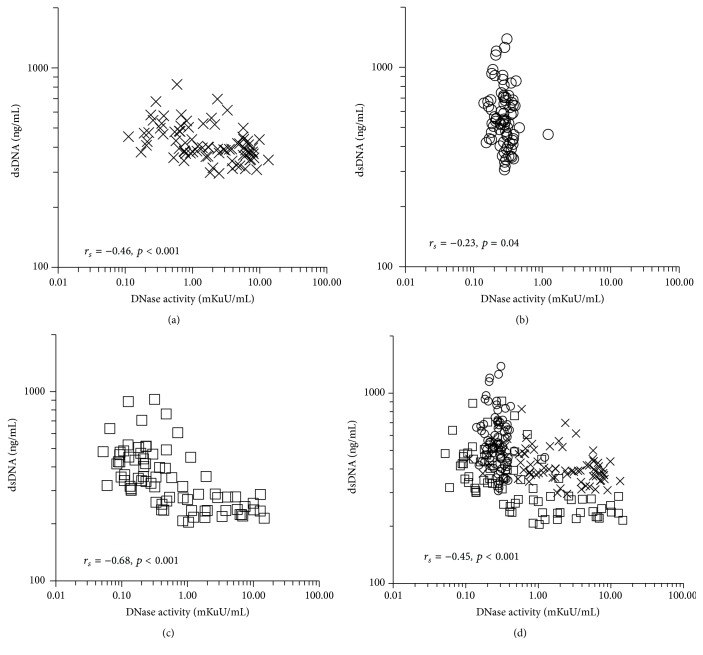
Relationship between serum double-strand DNA (dsDNA) concentration and the activity of deoxyribonuclease (DNase). Spearman's tests were performed at (a) baseline (*n* = 79), (b) immediately after surgery (PostOp, *n* = 79), and (c) the day after surgery (POD-1, *n* = 79). (d) When the data from all three time points (baseline, PostOp, and POD-1) were combined (merge), a moderate correlation between these two variables was observed (*r*_*s*_ = −0.45, *p* < 0.001). X marks, baseline; open circles, PostOp; open squares, POD-1. *r*_*s*_: correlation coefficient calculated from Spearman's tests.

Serum DNase activity was reduced in the entire cohort immediately after surgery. On POD-1, DNase activity varied widely, which suggested that postoperative recovery of DNase activity was affected by perioperative factors (Figure 2(a)). We also measured the antigen concentration of DNase-I but found no significant difference at each of the three time points (Figure 2(b)). Patients in the off-pump surgery group tended to show more rapid recovery of DNase activity than those in the on-pump surgery group (*p* = 0.016, Figure 2(c)).  Figure 3 shows the correlation between serum DNase activity and serum dsDNA concentration. When data from all three time points were combined (baseline, PostOp, and POD-1), a moderate negative correlation between these two variables was observed (*r*_*s*_ = −0.45, *p* < 0.001, Figure 3(d)). Samples with low DNase activity (<1 mKuU/mL) had significantly higher dsDNA concentration (median 472 ng/mL, IQR 380–585 ng/mL, and *n* = 163) compared with those with high DNase activity (≥1 mKuU/mL) (median 358 ng/mL, IQR 277–398 ng/mL, *n* = 74, and *p* < 0.001).

### 3.2. Correlation between Neutrophil Activation and dsDNA Concentration

**Figure 4:**
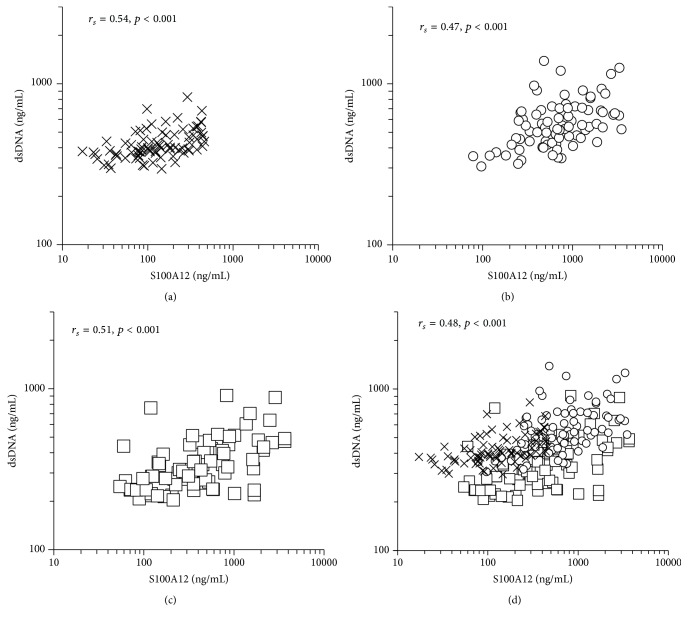
Association between the neutrophil activation marker S100A12 and serum double-strand DNA (dsDNA) concentration. (a) Baseline (*n* = 80), (b) immediately after surgery (PostOp, *n* = 80), and (c) the day after surgery (POD-1, *n* = 80). (d) When the data from all three time points (baseline, PostOp, and POD-1) were combined (merge), a moderate correlation between these two variables was observed (*r*_*s*_ = 0.48, *p* < 0.001). X marks, baseline; open circles, PostOp; open squares, POD-1. *r*_*s*_: correlation coefficient calculated from Spearman's tests.

**Figure 5:**
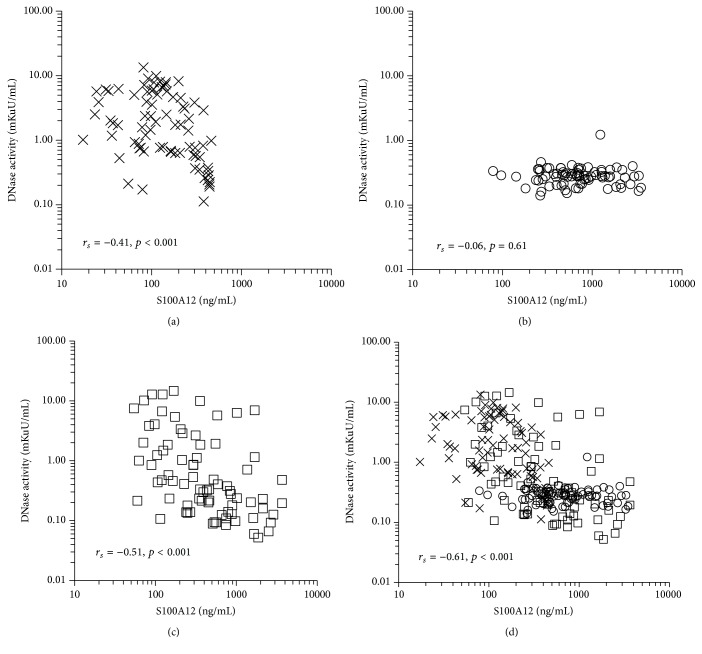
Relationship between the activity of deoxyribonuclease (DNase) and the neutrophil activation marker S100A12. (a) Baseline (*n* = 79), (b) immediately after surgery (PostOp, *n* = 79), and (c) the day after surgery (POD-1, *n* = 79). (d) When the data from all three time points (baseline, PostOp, and POD-1) were combined (merge), a moderate correlation between these two variables was observed (*r*_*s*_ = −0.61, *p* < 0.001). X marks, baseline; open circles, PostOp; open squares, POD-1. *r*_*s*_: correlation coefficient calculated from Spearman's tests.

To study the relationship between the dsDNA concentration and function of neutrophils, we measured S100A12 concentration, and the correlation between S100A12 and dsDNA concentration was examined. Concentrations of dsDNA were significantly correlated with S100A12 concentration at all three time points (*r*_*s*_ = 0.54, *p* < 0.001 at baseline, Figure 4(a); *r*_*s*_ = 0.47, *p* < 0.001 at PostOp, Figure 4(b); and *r*_*s*_ = 0.50, *p* < 0.001 on POD-1, Figure 4(c)). When the three measurements were combined, there was a significant correlation between S100A12 and dsDNA concentration (*r*_*s*_ = 0.48, *p* < 0.001, Figure 4(d)). S100A12 concentration showed a significant negative correlation with DNase activity at baseline (*r*_*s*_ = −0.41, *p* < 0.001, Figure 5(a)). PostOp concentration of S100A12 was elevated, DNase activity was reduced (Figure 5(b)), and S100A12 concentration on POD-1 varied widely, maintaining the negative correlation with DNase activity (*r*_*s*_ = −0.52, *p* < 0.001, Figure 5(c)). When the measurements from the three time points were combined, there was a significant negative correlation between S100A12 and DNase activity (*r*_*s*_ = −0.61, *p* < 0.001, Figure 5(d)), which suggested that activation of neutrophils resulted in an inhibitory effect on the activity of serum DNase.

### 3.3. Relationship between Serum dsDNA Concentration and Renal Function

**Figure 6:**
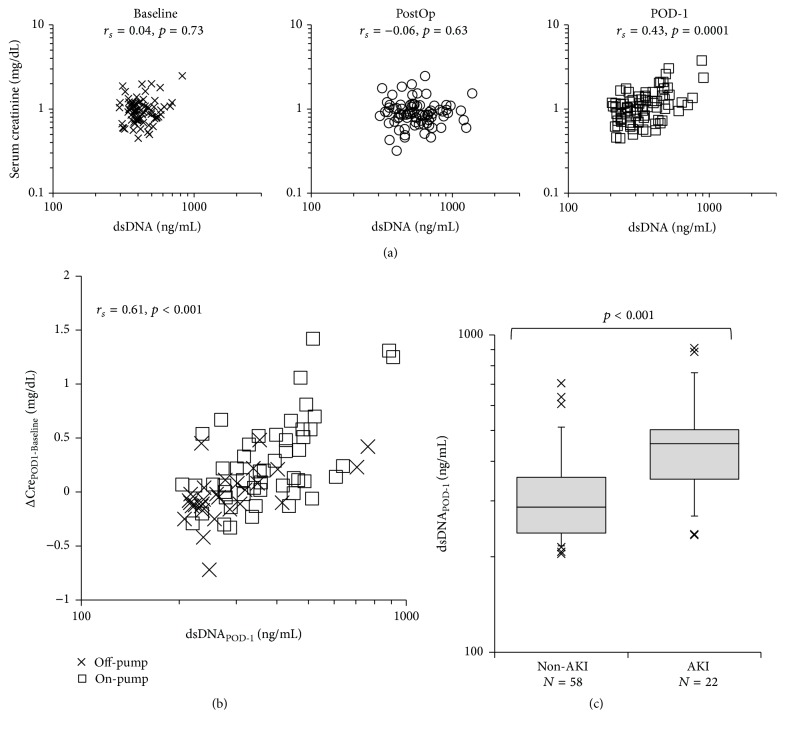
Association between serum double-strand DNA (dsDNA) concentration and serum creatinine concentration. (a) Relationship between serum dsDNA concentration and serum creatinine concentration. Spearman's tests were performed at baseline, immediately after surgery (PostOp), and the day after surgery (POD-1). X marks, baseline; open circles, PostOp; open squares, POD-1. (b) Relationship between the change in serum creatinine concentration from baseline to POD-1 (ΔCre_POD1-Baseline_) and dsDNA concentration on POD-1. X marks, off-pump surgery; open squares, on-pump surgery. (c) Serum dsDNA concentration in patients with or without acute kidney injury (AKI). Patients with AKI showed a significantly higher value of dsDNA on POD-1 than those without AKI (non-AKI). *r*_*s*_: correlation coefficient calculated from Spearman's tests.

Twenty-two patients were diagnosed with AKI (27.5%, three patients underwent off-pump surgery, and 19 patients underwent on-pump surgery). In one patient, renal replacement therapy was performed from POD-1 because of metabolic acidosis (stage 3 AKI), but all other patients with AKI had stage 1 impairment. Patients with postoperative AKI had higher baseline creatinine concentration than patients who did not develop AKI (*n* = 58). The duration of surgery was significantly longer (*p* < 0.001), and significantly more red cell transfusions were administered (*p* = 0.002) in those who developed AKI. Patients with AKI were found to have significantly higher serum IL-8 (*p* < 0.001), troponin-T (*p* = 0.02), and NT-proBNP (*p* = 0.04) concentrations immediately after surgery (Table 2). In the entire cohort, PostOp serum NGAL and creatinine concentrations on POD-1 were significantly correlated (*r*_*s*_ = 0.56, *p* < 0.001). Patients diagnosed with AKI exhibited significantly higher serum NGAL concentration immediately after surgery (median 136 ng/mL, IQR 81–218 ng/mL in patients with AKI compared with median 81 ng/mL, IQR 58–116 ng/mL in those without, *p* = 0.005).  Figure 6(a) shows the relationship between serum creatinine concentration and dsDNA concentration. By the end of surgery, serum creatinine concentration was not significantly elevated, despite elevation in dsDNA concentration. However, on POD-1, patients with high dsDNA concentration showed elevated creatinine concentration, and there was a significant association between these two variables (*r*_*s*_ = 0.43, *p* < 0.001, Figure 6(a)). There was also a significant correlation between the perioperative change in serum creatinine concentration between baseline and POD-1 (Δcreatinine) and dsDNA concentration (*r*_*s*_ = 0.61, *p* < 0.001, Figure 6(b)). Patients with AKI (median 455 ng/mL, IQR 351–503 ng/mL) had significantly higher dsDNA concentration than those without (median 287 ng/mL, IQR 238–356 ng/mL, and *p* < 0.001, Figure 6(c)). We analyzed correlation between Δcreatinine and possible confounding factors for the morbidity AKI, including baseline creatinine concentration, type of surgery (on-pump or off-pump), amount of red cell transfusion, PostOp concentrations of IL-8, troponin-T, and NT-proBNP, and duration of surgery. Surgical duration (*p* = 0.002) and dsDNA on POD-1 (*p* = 0.003) were significantly contributing factors to AKI (Table 3), and the AUROC of this logistic regression model was 0.86 for the discrimination of AKI. To determine the contribution of autoimmune antibodies to renal dysfunction, we measured anti-nucleosome antibody and pANCA concentration on POD-1. None of the patients showed positivity of these autoimmune antibodies.

## 4. Discussion

### 4.1. Perioperative Kinetics of Circulating dsDNA in Patients Undergoing Cardiac Surgery

We found that circulating dsDNA concentration was elevated postoperatively, and it fell by POD-1. These kinetics are similar to previously reported results from patients with multiple trauma [3]. Tissue damage, systemic inflammation, bleeding, and activation of hemostatic reactions are common characteristics of major surgery and trauma. Furthermore, recently published data showed that plasma histone levels were elevated both after severe trauma [14] and after pediatric cardiac surgery using cardiopulmonary bypass [15], which suggested that nucleosome components could be released immediately after cardiac surgery or trauma. Although dsDNA and histone could be released from multiple sources including damaged tissue and apoptotic cells, neutrophils could be an important source, because use of cardiopulmonary bypass circuit and reuse of suctioned blood could activate neutrophils and could promote NET formation after cardiac surgery. Nevertheless the mechanisms underpinning these kinetics consist of production and release of free dsDNA into the serum and degradation by DNase-I.

We found that serum free dsDNA concentration was significantly correlated with the neutrophil activation marker S100A12. This finding suggests a major contribution of neutrophil-derived dsDNA to elevation in serum dsDNA concentration, supporting our use of dsDNA concentration as a marker of NET release. Immediately after surgery, a wide range of dsDNA concentrations was observed in the context of low DNase activity (Figure 3(b)), possibly reflecting a large variation in NET formation (as dsDNA concentration showed a significant correlation with S100A12 concentration at that time point, Figure 4(b)).

With regard to degradation of dsDNA, we found that serum DNase activity was reduced immediately after surgery, but there was no change in the antigen concentration of DNase-I. There was a negative correlation between dsDNA concentration and DNase activity (Figure 3). These findings suggest that postoperative elevation in circulating dsDNA concentration could be partially explained by the reduction in DNase activity. Furthermore, patients undergoing off-pump surgery tended to recover DNase activity more quickly than patients undergoing cardiopulmonary bypass (Figure 3(c)). This recovery might be the cause of lower circulating dsDNA concentration. A reduction in serum DNase activity has been reported in systemic lupus erythematosus [16] and chronic inflammatory bowel disease [17]. Therefore, inflammation might reduce DNase activity, and recovery from inflammation might be an important factor for reducing circulating dsDNA concentration. Interestingly, DNase-I antigen concentration did not change during the study period, suggesting the existence of an inhibitory factor for DNase-I. Furthermore, serum S100A12 concentration was negatively correlated with DNase activity. These findings may indicate that DNase activity and dsDNA concentration are regulated by mechanisms associated with activation of neutrophils.

### 4.2. Potential Contribution of NETs to Perioperative Renal Dysfunction

The perioperative incidence of AKI was 27.5% in our cohort; all patients with AKI exhibited an elevation in serum creatinine concentration of >0.3 mg/dL from baseline to POD-1. These patients showed a significant elevation in PostOp serum NGAL concentration compared with patients without AKI. The perioperative change in serum creatinine concentration showed a moderate association with serum dsDNA concentration on POD-1. Additionally, serum dsDNA concentration on POD-1 was a significantly contributing factor to perioperative AKI in our cohort.

Although the exact mechanism could not be determined by the current study design, several mechanisms could be associated with this result. First, histone is one of the components of NETs, as well as dsDNA, and it could damage kidney tissue via toll-like receptor-2 and toll-like receptor-4 [18]. Second, granular antimicrobial proteins from neutrophils, including neutrophil elastase and myeloperoxidase, are also components of NETs, and they can damage endothelial tissue. Surgically induced elevation in NETs concentration could trigger endothelial dysfunction, and it might affect the microcirculation in renal tissue. Third, impairment of NET degradation is associated with renal dysfunction in some diseases, including systemic lupus erythematosus [16] and anti-neutrophil cytoplasmic antibody-associated vasculitis [19]. In these pathological conditions, inadequate degradation of NETs induces an autoimmune response to the residual NET components, including dsDNA and cytoplasmic proteins of neutrophils. Based on these facts, anti-nucleosomal antibody and pANCA were measured in this study, but neither of these autoimmune antibodies were positive, which suggested that none of these autoimmune responses contribute to the early phase of renal dysfunction. Nevertheless, based on the results of current study, further research will be necessary to study whether cell-free DNA or NETs induce kidney injury in more simplified experimental models and to illuminate the underlying pathophysiologic mechanisms.

### 4.3. Study Limitations

There are some limitations to this study. First, this study was designed as a reevaluation of a study for lung dysfunction after cardiac surgery, and patients with postoperative cardiac failure were excluded to eliminate cardiogenic pulmonary edema [10]. Therefore, the current results cannot be extrapolated to patients with postoperative cardiac failure. Second, in this study, we could not eliminate the sources of cell-free dsDNA other than neutrophils. The results of S100A12 suggested significant correlation between neutrophil activation and elevation in serum dsDNA concentration; however, we could not demonstrate whether the dsDNA bound neutrophil specific markers, such as myeloperoxidase and neutrophil elastase, because of sample shortage. Apoptosis and surgical destruction of tissues might be sources of dsDNA. At present, differentiation of the sources of dsDNA is difficult. Therefore, dsDNA from other sources might participate in the pathophysiology of perioperative organ dysfunction. Third, we could not eliminate the contribution of ischemia-reperfusion in the kidney because we could not detect intraoperative renal ischemia with high sensitivity. Although ischemia could cause neutrophil activation, we could not evaluate the relationship between ischemia and neutrophil activation. Multiple factors other than an elevation in serum dsDNA concentration could contribute to renal tissue damage, which could affect the relationship between the concentration of dsDNA and kidney function. Finally, the types of surgery were limited in the study cohort. Therefore, large-scale studies are required to determine the relationship between serum concentration of circulating dsDNA and perioperative renal dysfunction. Subgroup analyses for different types of surgery might also be helpful for determining the contribution of confounding factors.

## 5. Conclusions

Serum dsDNA concentration is elevated after cardiac surgery, which might be explained by activation of neutrophils. Prolonged elevation in circulating dsDNA concentration was associated with perioperative renal dysfunction. These results could help in identifying dsDNA as a new biomarker for postoperative risk assessment of patients with cardiac surgery. Further large-scale prospective studies will be needed to illuminate the relationship between circulating dsDNA concentration and perioperative renal dysfunction and the potential pathophysiologic mechanisms that might be responsible.

## Figures and Tables

**Table 1:** Patients' characteristics.

	Total	On-pump surgery	Off-pump surgery	*p *value
Number of patients	80	51	29	
				Chi-square test
Male/female (male%)	57/23 (71%)	34/17 (67%)	23/6 (79%)	0.92
				Kruskal-Wallis test
Age (year)	68 (58–74)	67 (53–74)	70 (60–74)	0.08
Body weight (kg)	60 (53–68)	59 (53–68)	61 (57–68)	0.54
White blood cell count (cells/mm^3^)	5840 (4850–7100)	5600 (4815–7100)	5950 (4900–6900)	0.70
Hemoglobin (g/dL)	12.7 (11.5–13.5)	12.4 (11.4–13.3)	13.1 (11.8–13.7)	0.13
Platelet count (10^4^/mm^3^)	19.1 (15.5–23.8)	18.5 (14.8–23.1)	20.7 (16.5–25.6)	0.21
Total protein (g/dL)	7.2 (6.6–7.4)	7.2 (6.7–7.3)	7.2 (6.6–7.6)	0.13
Albumin (g/dL)	4.1 (3.8–4.4)	4.2 (4.0–4.4)	4.0 (3.8–4.3)	0.21
BUN (mg/dL)	18.0 (14–22)	17.5 (14.0–21.9)	18.4 (15.4–22.0)	0.59
Creatinine (mg/dL)	0.95 (0.75–1.2)	0.92 (0.76–1.1)	0.97 (0.75–1.2)	0.83
Mean blood pressure (mmHg)	90 (82–98)	85 (78–96)	94 (87–103)	0.006
Heart rate (beats/min)	70 (62–78)	69 (62–77)	70 (61–79)	0.96
PaO_2_/F_i_O_2_ ratio	427 (331–500)	417 (342–500)	428 (327–490)	0.97
Duration of surgery (min)	385 (315–450)	405 (346–486)	320 (254–391)	0.0004
Duration of cardiopulmonary bypass (min)		197 (160–254)		
Transfusion				
Red cell (units^a^)	0 (0–4)	2 (0–4)	0 (0–2)	0.09
Fresh frozen plasma (units^a^)	0 (0–6)	0 (0–8)	0 (0–0)	0.001
Platelet (units^b^)	0 (0–0)	0 (0–20)	0 (0–0)	0.001

Values are median (interquartile range).

^a^One unit of packed red cells or fresh frozen plasma was derived from 200 mL of donated blood.

^b^One unit of packed platelet concentrate contained 2 × 10^10^ platelets.

BUN: blood urea nitrogen.

**Table 2:** Characteristics of patients with or without acute kidney injury.

	AKI	Non-AKI	*p* value
Number of patients	22	58	
			Chi-square test
Male sex	17 (77%)	40 (69%)	0.54
Diabetes mellitus	5 (22.7%)	20 (34.5%)	0.4
Type of surgery (on-pump/off-pump)	19/3	33/25	0.002
			Mann–Whitney *U *test
Age (year)	68 (61–74)	67 (56–74)	0.66
Body weight (kg)	62 (58–68)	60 (53–68)	0.31
Creatinine at baseline (mg/dL)	1.06 (0.88–1.20)	0.90 (0.74–1.11)	0.047
Data immediately after surgery			
White blood cell count (cells/mm^3^)	10300 (8658–12800)	9980 (7875–13153)	0.95
Platelet count (10^4^/mm^3^)	9.2 (8.2–10.5)	10.6 (7.8–14.6)	0.28
Albumin (g/dL)	3.5 (2.9–3.6)	3.2 (2.7–3.6)	0.22
PaO_2_/F_I_O_2_ ratio	245 (168–319)	279 (205–348)	0.17
Red cell transfusion (units^a^)	4 (2–4)	0 (0–4)	0.002
Serum interleukin-8 (pg/mL)	97 (41–173)	37 (2–62)	<0.001
Serum troponin-T (ng/mL)	0.70 (0.46–1.14)	0.39 (0.19–0.71)	0.02
Serum NT-proBNP (pg/mL)	329 (166–618)	137 (69–567)	0.04
Duration of surgery (min)	489 (400–580)	353 (295–410)	<0.001
Serum dsDNA concentration on POD-1 (ng/mL)	455 (351–503)	287 (238–356)	<0.001

^a^One unit of packed red cells or fresh frozen plasma was derived from 200 mL of donated blood.

AKI: patients diagnosed with acute kidney injury.

Non-AKI: patients without acute kidney injury.

POD-1: postoperative day-1 (the day after surgery).

OPCAB: off-pump coronary arterial bypass grafting.

NT-proBNP: N-terminal pro-B-type natriuretic peptide.

**Table 3:** Results of multiple logistic regression analyses for contributing factors to perioperative morbidity of acute kidney injury in the total study cohort.

	Odds ratio	*p* value
Duration of surgery (h)	1.60 (1.19–2.13)	0.003
Serum dsDNA on POD-1 (per 100 ng/mL)	2.09 (1.28–3.40)	0.002

dsDNA: double-strand DNA.

POD-1: postoperative day-1 (the day after surgery).
